# Investigation of the immunoregulatory mechanisms of total saponins from black ginseng

**DOI:** 10.29219/fnr.v70.13372

**Published:** 2026-02-05

**Authors:** Kuo Wang, Jiating Li, Liyan Huang, Mingran Luan, Chao Liu, Bao Zhong, Fenglin Li

**Affiliations:** 1 College of Food Science and Engineering, Changchun University, Changchun, P.R. China; 2 College of Food Science and Nutritional Engineering, Jilin Agriculture Science and Technology University, Jilin, P.R. China; 3 Brewing Technology Innovation Center of Jilin Province, Jilin, P.R. China; 4 School of Public Health, Jilin Medical University, Jilin, P.R. China; 5 School of Food Science and Engineering, Jilin Agricultural University, Changchun, P.R. China; 6 Culinary Nutrition and Premade Cuisine R&D Engineering Research Center, Jilin, P.R. China

**Keywords:** immunoregulatory, total saponins, black ginseng

## Abstract

**Objective:**

This study aimed to elucidate the immune-regulating effects and underlying mechanisms of action of total saponins extracted from black ginseng in an immunosuppressed murine model.

**Methods:**

The chemical composition of black ginseng total saponins (BGTS) was analyzed using high-performance liquid chromatography, which revealed a high content of rare ginsenosides, such as Rk1, Rg5, and Rg3. Immunosuppressed mice were administered BGTS, and key immunological parameters were assessed, including body weight, spleen and thymus indices, cytokine and immunoglobulin levels, and the expression of immune-related genes and proteins.

**Results:**

BGTS treatment significantly improved body weight and immune organ indices and promoted the secretion of cytokines, including interleukin (IL) 2, IL-1β, tumor necrosis factor-alpha, immunoglobulin (Ig) A, IgG, and IgM. Mechanistically, BGTS significantly activated the Toll-like receptor 4 (TLR4)/myeloid differentiation factor 88/nuclear factor-κB signaling pathway at the protein level and upregulated gene expression of TLR-4, TNF-α, IL-6, and IL-1β.

**Conclusion:**

These findings suggest that BGTS exerts notable immunomodulatory effects by enhancing innate immune responses, primarily by activating the TLR4-mediated signaling pathway. Further studies are necessary to isolate the contribution of individual ginsenosides and evaluate the long-term safety and clinical potential of BGTS.

## Popular scientific summary

- Black ginseng total saponins (BGTS) are rich in rare ginsenosides, including Rk1, Rg5, and Rg3, which are considered key bioactive components contributing to their biological functions.
- In an immunosuppressed mouse model, BGTS markedly improved immune function by increasing body weight, enhancing spleen and thymus indices, and promoting the production of immune-related cytokines and immunoglobulins.
- BGTS stimulated innate immune responses by activating the Toll-like receptor 4 (TLR4)/MyD88/NF-κB signaling pathway and upregulating the expression of pro-inflammatory cytokine genes.
- These findings highlight the potential of BGTS as a natural immunomodulatory agent and provide a scientific basis for its further development in immune health–related applications.

Immune regulation plays a critical role in maintaining health and defending against diseases (1, 2). Dysregulation of the immune system is closely linked to various pathological conditions, including inflammation, tumors, and autoimmune disorders (3). Consequently, the development of drugs that can safely and effectively modulate the immune function has become a prominent focus in modern pharmaceutical research. In recent decades, there has been a growing emphasis on the exploration of natural products as sources of novel immunoregulatory agents (4). These compounds often exhibit a broad spectrum of biological activities with relatively low toxicity, making them attractive candidates for drug development. Among them, plant-derived saponins have drawn considerable attention because of their diverse pharmacological properties, including anti-inflammatory, antitumor, and immunomodulatory effects (5).

Ginseng, a traditional and highly valued Chinese medicinal herb, is widely recognized for its immune-enhancing, qi-boosting, and body-strengthening properties (6, 7). Saponins are regarded as the key active components responsible for the pharmacological effects of ginseng, with particularly notable immune-regulatory activity (8, 9). Research has shown that ginsenosides can effectively activate macrophages, enhance their phagocytic function, and stimulate the secretion of pro-inflammatory cytokines, such as tumor necrosis factor-α (TNF-α), interleukin-6 (IL-6), and interleukin-1β (IL-1β) (10, 11). Black ginseng, a processed form of Panax ginseng obtained through repeated steaming and drying cycles, has been traditionally used in East Asian medicine for its enhanced medicinal properties compared with white or red ginseng. The steaming process alters the chemical composition of ginseng, leading to an increased concentration of specific saponins known as ginsenosides, which undergo substantial changes in their chemical composition, particularly with an increase in rare ginsenosides such as Rg3, Rk1, and Rg5 (12, 13). These compounds endow black ginseng with superior biological activities compared with white ginseng, including enhanced antioxidant, anti-fatigue, and antitumor effects (14). Studies have demonstrated that ginsenoside Rg3, abundant in black ginseng, can regulate the expression of inflammatory factors via signaling pathways, such as the nuclear factor kappa B (NF-κB) pathway (15, 16). However, most studies have focused on individual ginsenoside components or examined the overall immune phenotypes. Toll-like receptor 4 (TLR4) and its downstream signaling pathway, myeloid differentiation factor 88 (MyD88)/NF-κB, serve as critical intermediaries that link immune recognition to the expression of inflammatory factors (17, 18). For black ginseng total saponins (BGTS), a complex mixture of components, the precise mechanism by which they target specific pattern recognition receptors, such as TLR4, on immune cells and activate downstream signaling pathways such as MyD88/NF-κB, remains unclear. Additionally, it is unknown whether BGTS activates this pathway to coordinate the regulation of various immune effector molecules at both gene transcription and protein expression levels. The lack of experimental evidence supporting this causal pathway highlights the need for further investigation.

This study aimed to investigate the immunomodulatory role of BGTS in enhancing systemic immune responses using a non-disease-specific animal model, thereby confirming its broad immunomodulatory properties. Experiments elucidate how BGTS activates the TLR4/MyD88/NF-κB signaling pathway, thereby upregulating the transcriptional levels of key inflammatory factors, enhancing their protein synthesis and release, and ultimately coordinating the immune response. These results provide a solid pharmacological basis for a more comprehensive understanding of the immunomodulatory effects of total saponins in black ginseng.

## Materials and methods

### Preparation of black ginseng saponins

Black ginseng powder was accurately weighed and extracted with 60% ethanol at a solid-to-liquid ratio of 1:20 (g/mL). A compound enzyme preparation consisting of cellulase (Shanghai Yien Chemical Technology Co., Ltd., Shanghai, China) and pectinase (Shanghai Yuanye Bio-Technology Co., Ltd., Shanghai, China) in a 2:1 mass ratio was added for enzymatic hydrolysis. After the enzymatic reaction was complete, the mixture was heated in a water bath at 90°C for 10 min to inactivate the enzymes. The solution was then centrifuged at 8,000 r/min for 15 min, and the supernatant was collected. The residue was re-dissolved in 60% ethanol (solid-to-liquid ratio of 1:20), subjected to ultrasonic treatment at 400 W and 40°C, and centrifuged again. The two supernatants were combined and concentrated under reduced pressure to obtain a crude extract of black ginseng.

The concentrate was dissolved in an appropriate amount of deionized water and subjected to liquid–liquid extraction with water-saturated n-butanol. The organic phase was collected three times, combined, and concentrated under reduced pressure at 45°C until no solvent remained. The crude extract was purified using AB-8 macroporous adsorption resin. Initially, a large volume of deionized water was used to remove water-soluble impurities (e.g. sugars), followed by washing with 30% (v/v) ethanol to eliminate weakly polar impurities. Finally, 70% (v/v) ethanol was used to elute saponins. The eluate was collected, concentrated under reduced pressure, and dried to obtain the total saponin content of black ginseng.

### Determination of saponin content

Saponin standards, including Rg1, Re, Rb1, Rc, Rb2, S-Rg2, R-Rg2, Rh1, Rd, S-Rg3, R-Rg3, Rk1, Rg5, Ck, and Rh2 (purchased from Chengdu Biopurify Phytochemicals Ltd., Chengdu, China), were accurately weighed and dissolved in chromatographic-grade methanol to prepare standard stock solutions at a concentration of 500 µg/mL. The solutions were filtered through a 0.22 μm membrane filter before use. Working solutions were prepared by appropriately diluting the stock solutions. Saponin content was determined using high-performance liquid chromatography. Chromatographic separation was performed on an Eclipse Plus Rapid Resolution HT C18 column (4.6 × 150 mm, 1.8 μm). The mobile phase consisted of 0.05% aqueous phosphoric acid and acetonitrile. The flow rate was set at 0.3 mL/min, the injection volume was 10 μL, and detection was conducted using a UV detector at 203 nm (19).

### Animal experiment

Forty male ICR mice (purchased from Changchun Yisi Laboratory Animal Technology Co., Ltd.) with an initial body weight of 20 ± 2 g were housed under standard laboratory conditions (temperature: 23 ± 2°C; humidity: 55 ± 5%; 12 h light/dark cycle) with free access to food and water. The animals were acclimatized for 1 week before the experiment. The mice were randomly divided into four groups (*n* = 10 per group): Normal Control (NC), Model Control (MC), Transfer Factor Oral Solution (TFOS), and BGTS (120 mg/kg). Except for the NC group, all mice were intraperitoneally injected with dexamethasone (4.1 mg/kg) once daily for seven consecutive days to induce immunosuppression. After successful model establishment, the NC and MC groups received distilled water via gavage, the Positive Control group was administered a TFOS, and the BGTS group received BGTS. Body weights were recorded every 4 days. All treatments were administered by oral gavage for 28 consecutive days. At the end of the experiment, the mice were sacrificed, and blood was collected for serum separation through centrifugation. Liver, spleen, and thymus tissues were harvested and stored at −80°C for further analysis.

### Measurement of blood indicators

Firstly, 50 μL of different concentrations of the standard was added to each standard well, and 10 μL of the sample to be tested was added to each sample well, followed by 40 μL of the sample diluent. Except for the blank wells, 100 μL of horseradish peroxidase-labeled detection antibody was added to each standard and sample well. The reaction wells were sealed with sealing film and incubated at 37°C in a water bath or incubator for 60 min. The liquid was discarded, the absorbent paper was patted dry, each well was filled with washing buffer and let stand for 1 min, and the washing buffer was discarded and patted dry on absorbent paper. The washing process was repeated five times. A 50 μL each of substrate was added to each well and incubated at 37°C in the dark for 15 minutes. Then, 50 μL of stop solution was added to each well. Within 15 min, the cells were incubated at 450 nm wavelength. The OD value of each well was measured at a wavelength, a standard curve was plotted, and the results were calculated. The enzyme-linked immunosorbent assay kit used included TNF-α, immunoglobulin M (IgM), interleukin-2 (IL-2), immunoglobulin G (IgG), interleukin-1β (IL-1 β), and immunoglobulin A (IgA) (Jiangsu Jingmei Biotechnology Co., Ltd., Nanjing, Jiangsu, China).

### Western blot analysis

Total protein was extracted from liver tissue using RIPA lysis buffer (Beijing Soluble Biotechnology Co., Ltd., Beijing) containing a mixture of protease and phosphatase inhibitors. Total protein concentration was determined according to the CBA kit instructions. After denaturation, the protein samples were separated using 10% sodium dodecyl sulfate-polyacrylamide gel electrophoresis and transferred onto a polyvinylidene fluoride membrane. The membrane was blocked with blocking buffer at room temperature for 1 h, then incubated with primary antibodies, such as TLR-4, MyD88, NF-κB, and β-actin (ImmunoWay Biotechnology Company, Suzhou, Jiangsu), at 4°C for 12 h. The membrane was washed five times with Tris-buffered saline buffer and incubated with a horseradish peroxidase-labeled secondary antibody at room temperature for 1 h. Finally, membranes were washed with Tris-buffered saline containing Tween 20 for 5 min. The results were developed and photographed using an enhanced chemiluminescence imaging system with enhanced chemiluminescence. The gray values of the bands on the nitrocellulose membranes were compared and analyzed using Celina image analysis software (20).

### Gene expression analysis

mRNA was isolated from the liver tissue using the TRIzol method. After purity testing, the first-strand cDNA was synthesized using the FastKing cDNA First-Strand Synthesis Kit (Beijing Tiangen Biotech Co., Ltd.). Quantitative real-time polymerase chain reaction was performed using a Talent qPCR PreMix (SYBR Green) kit (Beijing Tiangen Biotech Co., Ltd.). The primer sequences used in this study are listed in Table 1.

**Table 1:** Primer sequence of the genes

Gene	Primer	Sequence (5’-3’)
*TLR-4*	Forward primer	5’-TATTCGGCTATGACTGGGCACA-3’
	Reverse primer	5’-GATGGATACTTTCTCGGCAGGA-3’
*TNF-α*	Forward primer	5’-TGACAAGCCTGTAGCCCACG-3’
	Reverse primer	5’-TTGTCTTTGAGATCCATGCCG-3’
*IL-1β*	Forward primer	5’-GCACACCCACCCTGCA-3’
	Reverse primer	5’-ACCGCTTTTCCATCTTCTTCTT-3’
*IL-6*	Forward primer	5’-GAGGATACCACTCCCAACAGACC-3’
	Reverse primer	5’-CAGGTCTGTTGGGGAGTGG-3’
*β-actin*	Forward primer	5’-AGCCTTCCTTCTTGGGTATGG-3’
	Reverse primer	5’-CACTTGCGGTGCACGATGGAG-3’

Notes: Toll-like receptor 4 (*TLR-4*), Tumor necrosis factor-alpha (*TNF-α*), Interleukin-1 beta (*IL-1β*), Interleukin-6 (*IL-6*).

### Statistical analysis

All data are presented as mean ± standard deviation (SD). Statistical analysis was performed using SPSS software (IBM Corp., Armonk, NY, USA) to assess the differences between groups. One-way analysis of variance (ANOVA) with compare means was used to assess the significance of differences between groups, with significance set at *P* < 0.05 or *P* < 0.01. Different letters (a, b, and c) were used to indicate statistically significant differences between groups, where ‘a’ represents the highest value and ‘c’ represents the lowest value.

## Results

### Determination of ginsenosides in black ginseng

**Fig. 1:**
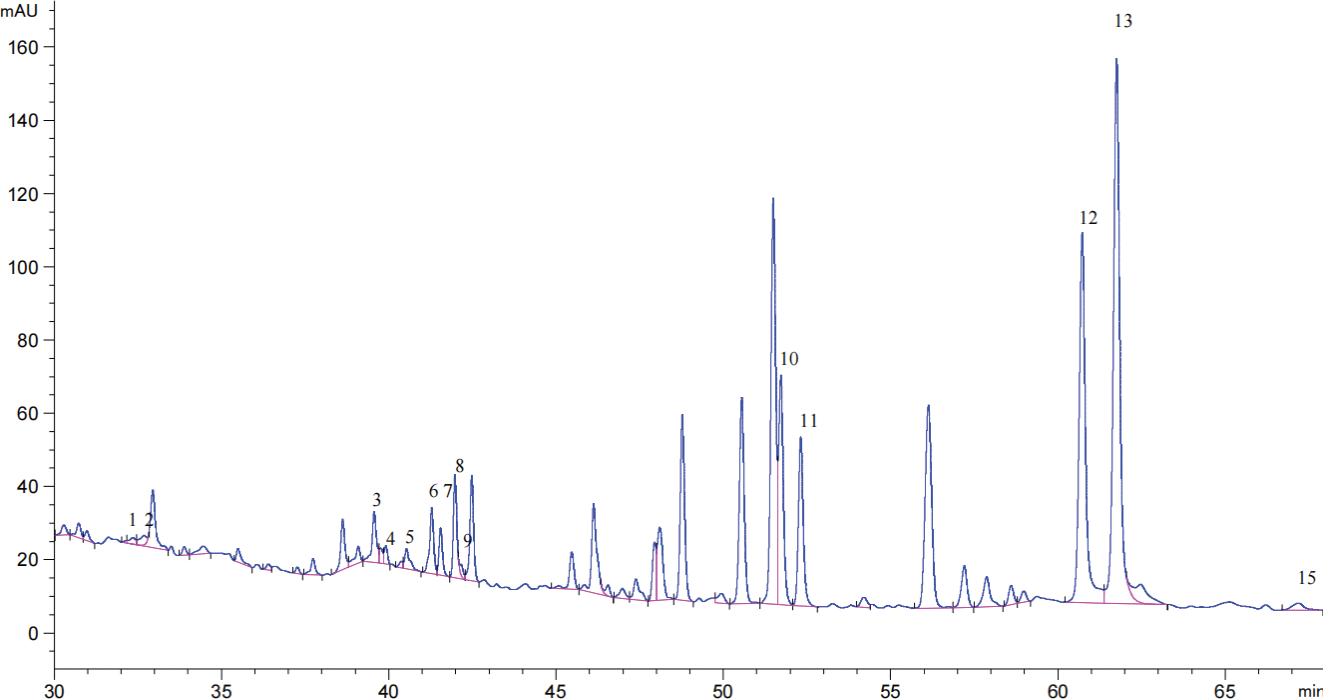
HPLC chromatogram of total ginsenosides extracted from black ginseng.

**Fig. 2:**
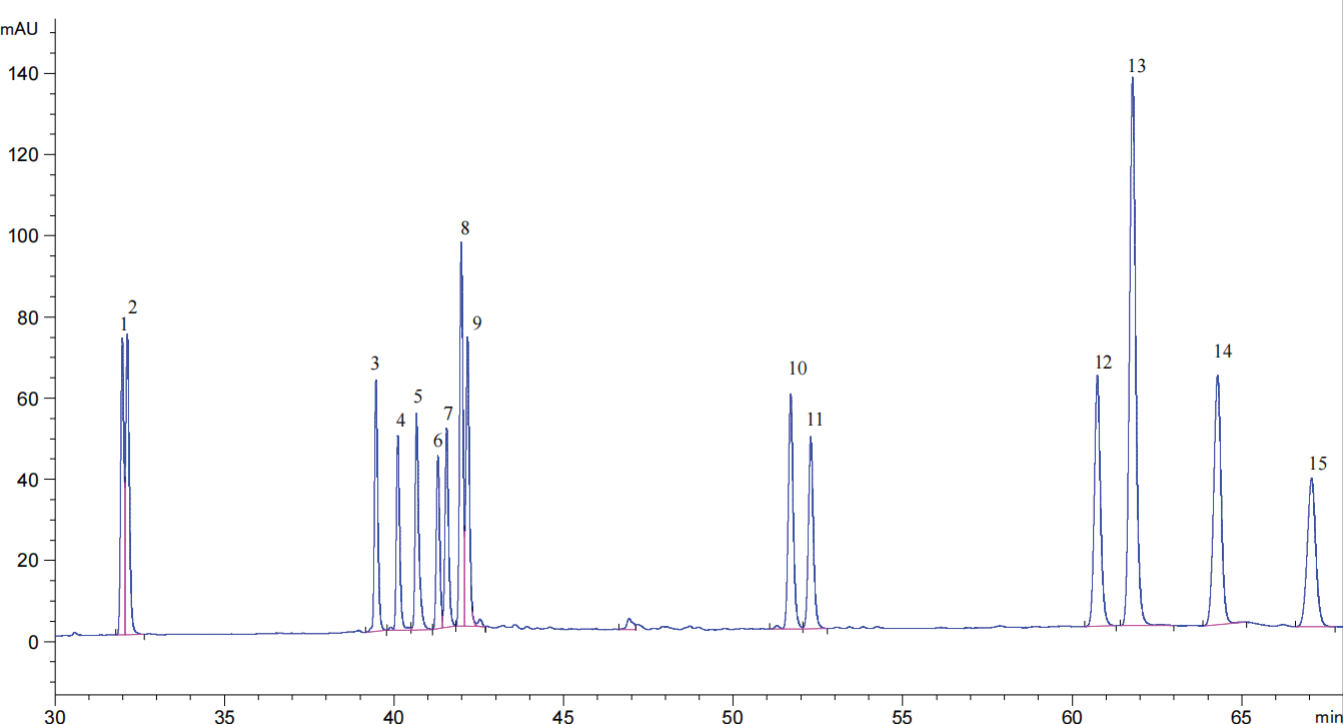
HPLC (High Performance Liquid Chromatography) chromatogram of ginsenoside standards. Peak 1: Ginsenoside Rg1, Peak 2: Ginsenoside Re, Peak 3: Ginsenoside Rb1, Peak 4: Ginsenoside Rc. Peak 5: Ginsenoside Rb2, Peak 6: Ginsenoside S-Rg2, Peak 7: Ginsenoside R-Rg2, Peak 8: Ginsenoside Rh1, Peak 9: Ginsenoside Rd, Peak 10: Ginsenoside S-Rg3, Peak 11: Ginsenoside R-Rg3, Peak 12: Ginsenoside Rk1, Peak 13: Ginsenoside Rg5, Peak 14: Ginsenoside Ck, and Peak 15: Ginsenoside Rh2.

As shown in Fig. 1, the total ginsenoside content in black ginseng extract reached 3351.38 μg/mL, based on quantitative analysis compared with the standard reference ginsenosides shown in Fig. 2. The major ginsenosides identified and their respective concentrations were as follows: Rg1 (29.42 μg/mL), Re (40.14 μg/mL), Rb1 (147.35 μg/mL), Rc (51.26 μg/mL), Rb2 (71.38 μg/mL), S-Rg2 (250.04 μg/mL), R-Rg2 (128.05 μg/mL), Rh1 (156.68 μg/mL), Rd (18.12 μg/mL), S-Rg3 (511.75 μg/mL), R-Rg3 (470.98 μg/mL), Rk1 (869.99 μg/mL), Rg5 (569.35 μg/mL), and Rh2 (36.80 μg/mL).

### Effects of total ginsenosides from black ginseng on body weight and immune organ indices in mice

**Fig. 3:**
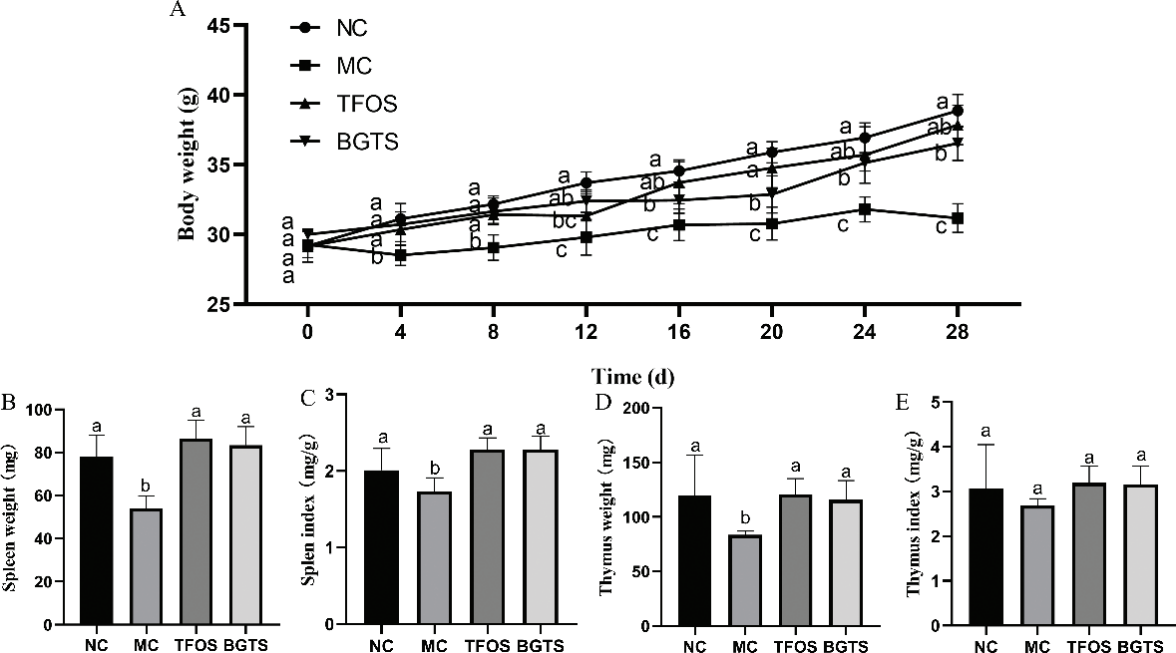
Body weight and immune organ indices of mice. (A) Changes in body weight during the experimental period, (B) Spleen weight, (C) Spleen index, (D) Thymus weight, and (E) Thymus index. Different superscript letters (a, b, and c) indicate statistically significant differences between groups (*P* < 0.05) according to Duncan’s multiple range test.

As shown in Fig. 3A, the body weight of mice in the MC group was significantly lower than that in the NC, TFOS, and BGTS groups, indicating that both TFOS and BGTS treatments effectively alleviated weight loss in immunosuppressed mice. The spleen weight results are shown in Fig. 3B. Compared with the MC group, spleen weights were significantly increased in the NC, TFOS, and BGTS groups, with no significant differences observed among these three groups. Similarly, the spleen index (Fig. 3C) was significantly elevated in the NC, TFOS, and BGTS groups compared with that in the MC group. As shown in Fig. 3D, thymus weight was significantly higher in the NC, TFOS, and BGTS groups than in the MC group, with no significant differences among the NC, TFOS, and BGTS groups. The thymus index (Fig. 3E) also showed an upward trend in the NC, TFOS, and BGTS groups compared with the MC group; however, the differences were not statistically significant.

### Effects of total ginsenosides from black ginseng on serum biochemical parameters in mice

**Fig. 4:**
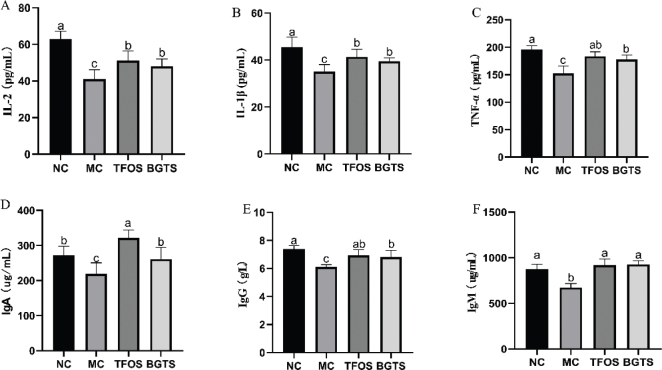
Serum biochemical indicators in mice. (A) IL-2 levels in serum, (B) IL-1β levels in serum, (C) TNF-α levels in serum, (D) IgA levels in serum, (E) IgG levels in serum, and (F) IgM levels in serum. Different superscript letters (a, b, and c) indicate statistically significant differences between groups (*P* < 0.05) according to Duncan’s multiple range test.

The effects of BGTS on serum biochemical indicators are shown in Fig. 4. Compared with the MC group, the levels of IL-2 were significantly increased in both the TFOS and BGTS groups (Fig. 4A). Similarly, IL-1β and TNF-α levels were significantly elevated in the TFOS and BGTS groups compared with the MC group (Fig. 4B and 4C). The serum immunomodulatory indicator IgA was significantly increased in both treatment groups relative to that in the MC group (Fig. 4D). As shown in Fig. 4E, the IgG levels in the TFOS and BGTS groups were significantly higher than those in the MC group. No significant differences were observed between the TFOS and NC groups. Additionally, IgM levels in the TFOS and BGTS groups were significantly higher than those in the MC group (Fig. 4F), with no significant differences observed between the treatment and NC groups.

### Effects of total ginsenosides from black ginseng on TLR-4/MyD88/NF-κB protein expression in mice

**Fig. 5:**
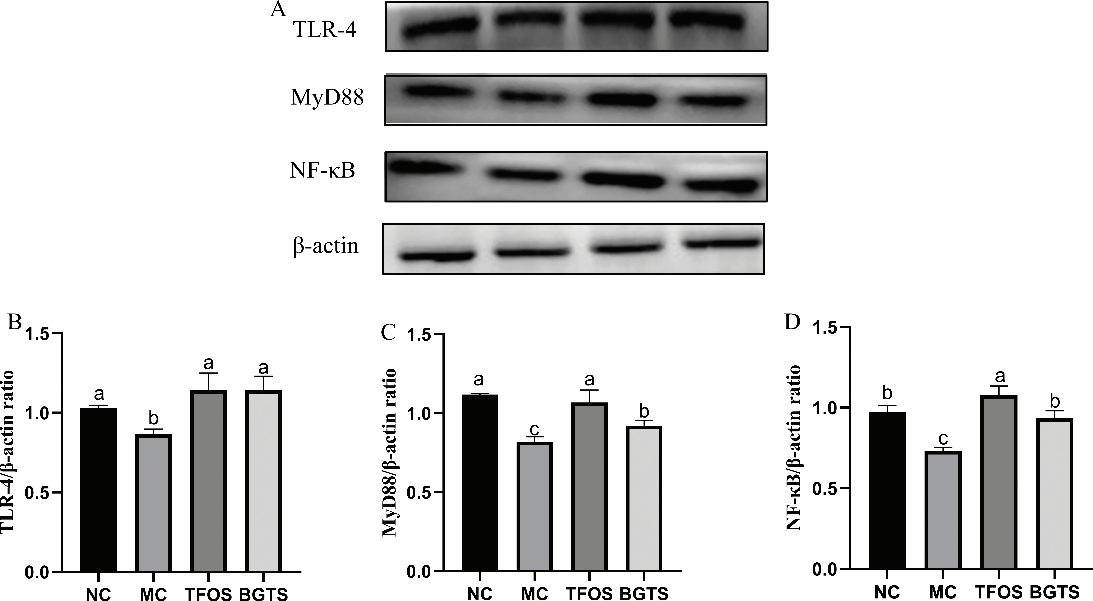
Expression of immunoregulatory proteins. (A) Representative western blot images of TLR-4, MyD88, and NF-κB protein expression, (B) Grayscale analysis of TLR-4 protein expression, (C) Grayscale analysis of MyD88 protein expression, (D) Grayscale analysis of NF-κB protein expression. Differences between the superscript letters (a, b, and c) in the graphs were statistically significant (*P* < 0.05) using Duncan’s Multiple Extreme Difference test.

The expression levels of immunoregulatory proteins are shown in Fig. 5. Compared with the MC group, the expression of TLR-4 protein was significantly increased in both the TFOS and BGTS groups, with no significant differences compared with the NC group. Similarly, the expression levels of MyD88 and NF-κB proteins were significantly upregulated in the TFOS and BGTS groups relative to the MC group. The expression of NF-κB protein showed no significant difference compared with the NC group.

### Effect of BGTS on the expression of TLR-4, TNF-α, IL-6, and IL-1β genes in mice

**Fig. 6:**
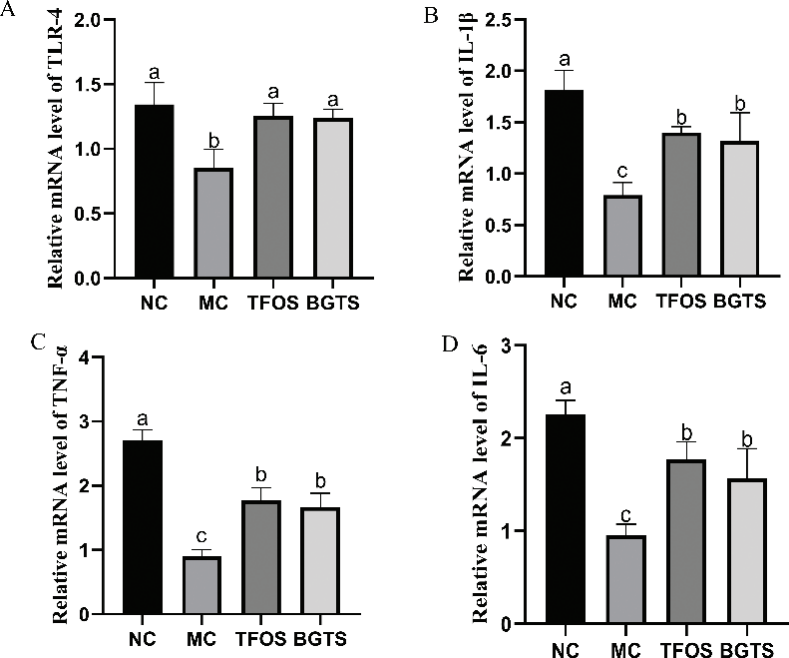
Results of immune regulation-related gene expression. (A) Relative mRNA level of *TLR-4*, (B) Relative mRNA level of *IL-1β*, (C) Relative mRNA level of *TNF-α,* and(D) Relative mRNA level of *IL-6*. Differences between the superscript letters (a, b, and c) in the graphs were statistically significant (*P* < 0.05) using Duncan’s Multiple Extreme Difference test.

The gene expression results are shown in Fig. 6. Compared with the MC group, the expression of *TLR-4* was significantly increased in the TFOS and BGTS groups, with no significant differences observed compared with the NC group. Compared with the MC group, the expression of *TNF-α*, *IL-6*, and *IL-1β* was significantly increased in the TFOS and BGTS groups, with no significant difference between the TFOS and BGTS groups.

## Discussion

In this study, we investigated the immunomodulatory effects of total saponins extracted from BGTS in a murine model, focusing on systemic biochemical parameters, immune organ indices, and relevant signaling pathways. The quantitative analysis revealed that BGTS contained a high total ginsenoside concentration (3351.38 μg/mL), with Rk1 (869.99 μg/mL), S-Rg3 (511.75 μg/mL), R-Rg3 (470.98 μg/mL), and Rg5 (569.35 μg/mL) as the major components. These ginsenosides, particularly the less polar types formed during steaming and aging, have been previously reported to possess strong bioactivities, including anti-inflammatory and immunostimulatory effects (19, 21, 22).

Immunosuppression is characterized by impaired cytokine production, reduced lymphocyte activity, and diminished immune organ function, leading to increased susceptibility to infection and reduced systemic immunity (23). In this context, cytokine dysregulation, particularly reductions in IL-2, IL-1β, and TNF-*α*, plays a pivotal role in weakening innate and adaptive immune responses (24). Therefore, restoring cytokine balance is a critical therapeutic strategy for reversing immunosuppressed conditions (25). In this study, BGTS significantly improved the immune status of the immunosuppressed mice, providing multiple lines of evidence for its immunoregulatory activity. The BGTS treatment restored body weight, spleen and thymus mass, and immune organ indices, suggesting improved immune organ development and recovery from external-stress-induced immunosuppression. These improvements were consistent with the biological actions of ginseng-derived saponins previously reported in models of immune dysfunction (26).

Serum cytokine analysis further demonstrated that BGTS effectively reversed cytokine dysregulation. IL-2, IL-1β, and TNF-α, key mediators of T-cell proliferation, macrophage activation, and inflammatory signaling (27, 28), were significantly elevated following BGTS administration. Since immunosuppression is commonly associated with the reduced expression of these cytokines, their restoration indicates that BGTS may enhance both innate and adaptive immune responses. In parallel, serum immunoglobulin levels (IgA, IgG, and IgM) were markedly increased, with IgA and IgG levels approaching or surpassing those in the NC group. This suggests that BGTS effectively stimulates humoral immunity and supports antibody-mediated host defense (29, 30).

At the molecular level, BGTS activates the TLR-4/MyD88/NF-κB signaling cascade, a central pathway in innate immunity that regulates cytokine production and inflammatory responses (31, 32). Increased expression of TLR-4, MyD88, and NF-κB proteins, together with upregulated transcription of TLR-4, TNF-α, IL-6, and IL-1β, demonstrates that BGTS exerts its immunomodulatory effects by activating innate immune receptors and downstream inflammatory signaling. Previous studies have shown that ginsenosides, such as Rg3, Rg5, and Rk1, modulate macrophage phagocytosis and dendritic cell maturation via similar pathways (21, 33, 34). Therefore, the immunoregulatory activity of BGTS is likely mediated by the synergistic action of multiple ginsenoside components acting on the TLR-4/MyD88/NF-κB axis.

Collectively, these findings indicate that BGTS effectively reverses immunosuppression by restoring cytokine production, promoting immunoglobulin synthesis, and activating key immune signaling pathways. This suggests that BGTS may have therapeutic potential as a natural immunomodulator or adjuvant in clinically relevant immunocompromised conditions, such as chemotherapy-induced immune suppression, chronic infections, or age-related immune decline. Furthermore, this study provides foundational *in vivo* evidence of the immunoregulatory properties of BGTS, which will serve as a basis for future mechanistic studies using specific cell models or disease-oriented experimental systems.

## Conclusion

This study demonstrates that total saponins extracted from black ginseng have significant immunomodulatory effects on immunosuppressed mice. Chemical profiling revealed that BGTS contains a high content of rare ginsenosides, such as Rk1, Rg5, and Rg3, which are known for their potent biological activities. *In vivo* experiments showed that BGTS effectively improved body weight, spleen and thymus indices, and promoted the secretion of key immune cytokines, such as IL-2, IL-1β, and TNF-α, and immunoglobulins, such as IgA, IgG, and IgM. Furthermore, BGTS significantly activated the TLR-4/MyD88/NF-κB signaling pathway at the protein level and significantly activated *TLR-4*, *TNF-α*, *IL-6*, and *IL-1β* gene expression levels, suggesting that its immunomodulatory mechanism is closely associated with the activation of innate immune pathways. Further research is necessary to explore the specific roles of individual saponins, to verify the long-term safety and efficacy of BGTS in clinical settings, and to conduct *in vitro* activity studies.
